# Beware of Strangers: Dogs’ Empathetic Response to Unknown Humans

**DOI:** 10.3390/ani14142130

**Published:** 2024-07-22

**Authors:** Micael M. Rivera, Julia E. Meyers-Manor

**Affiliations:** Ripon College, Ripon, WI 54971, USA

**Keywords:** canine, social behavior, emotional contagion, empathy

## Abstract

**Simple Summary:**

While dogs will rescue their owners in distress, no study has investigated whether or not dogs may do the same for strangers. This study explores this question by recording dogs’ behaviors, heart rate variability, and approach in response to a trapped calm or distressed stranger. The results show that whether the unfamiliar human was distressed or not did not affect whether the dog opened the door. Additionally, there was no difference in heart rate variability, how they approached the stranger through the door, or time near the door between dogs who were presented with a distressed or calm stranger. Dogs that did open the door to approach the unfamiliar human were described as less fearful by their owners. Dogs that opened also showed less aggression and fear when they approached the human behind the trapped door compared to dogs that did not open. These results show that dogs may not be able to show emotional contagion or respond differentially to distressed unknown humans. It also may be vital for dog owners to be present in order for complex behaviors such as emotional contagion and helping to occur towards strangers.

**Abstract:**

Empathy is a complex cognitive ability that has been studied in many social animals, including dogs. Previous studies have found that dogs would rescue their distressed owner more quickly than a calm owner and that dogs respond physiologically and behaviorally to the sound of crying strangers. However, no studies have explored the empathetic and emotional contagion capabilities of dogs towards strangers in rescue paradigms. In the present study, a stranger was placed behind a clear door and was told to cry (distress) or hum (neutral). The dogs’ door opening, stress behaviors, tone of approach, and physiological responses were measured. Dogs did not open more frequently or more quickly for the stranger in the distressed condition compared to the neutral condition. Additionally, there was no significant difference between the behavioral or physiological indicators of stress across conditions. It was also found that non-openers were reported by owners to have more fear and, in the empathy test, were more aggressive and fearful in their tone of approach. These results suggest that dogs may be less likely to exhibit empathy-like behaviors to unknown humans in an unfamiliar environment and that owners may be necessary to moderate a dog’s stress to show empathetic behaviors.

## 1. Introduction

Empathy, in its simplest form, refers to the sharing or recognition of another’s emotional state, often termed emotional contagion [1]. Empathy has been proposed to provide social cohesion and cooperation to groups of humans and animals (for reviews, see [2,3,4]). The search for empathy in animals helps to elucidate both evolutionary origins of empathy [5] and has welfare implications for animals [6]. Dogs, in particular, provide an interesting model in which to study the evolution of emotional contagion and empathy in animals because they are phylogenetically distant from humans but highly social with a long and close relationship with humans, which may have produced particularly complex social skills [7,8,9].

Although researchers have often disagreed on the requirements and organization of empathy (for a review, see [10]), three components of empathy are often discussed: matching with others (e.g., emotional contagion), understanding of others (e.g., perspective taking), and prosociality (with these elements combined either hierarchically [11] or in parallel [12]). Dogs have been studied in a variety of contexts with both humans and other dogs on their ability to display these components of empathy. Dogs show matching of others emotional states in studies of contagious yawning to humans ([13,14,15,16]; but see [17,18]), play contagion towards dogs [19], and contagion of distress behaviors or vocalizations to both dogs [20,21] and humans [21,22,23]. Dogs also show at least some understanding of others based on pointing studies [24,25], visual attention states [26], and studies where dogs understand the needs of their partner [27,28,29] (as reviewed in [10,30]). Finally, dogs exhibit prosociality in helping conspecifics to access food [31,32] and open doors for humans [33], although dogs certainly do not always show helping [34]. Recently, dogs have been studied for prosocial helping behavior in rescue paradigms [35,36,37], with most studies involving the owner of the dog trapped behind a door of an enclosure while the owner expressed distress (crying) or was in a neutral state (humming [35] or reading [36,37]). Dogs appear to show the prosocial elements of empathy towards individuals in distress, but these tests have primarily examined responses to their owners.

Arguments for and against empathetic explanations of dogs’ behavior in past studies have often hinged on comparisons to aspects of empathy often found in humans ([14,15]; for criticisms, see [10,38]). Research in humans has found that empathy is typically stronger for people with whom we are familiar than for those who are unfamiliar [39,40,41]. This familiarity bias has also been reported in chimpanzees [42,43] and dogs [15,44]. However, the research on empathy in humans and animals has not always found evidence of a familiarity bias. In humans, there may be differences in the presence of familiarity bias depending on the valence of the emotion (positive or negative). Motomura et al. [45] found that there was an empathetic response to strangers (i.e., a lack of familiarity bias) for negative emotional states but not positive emotions. In the case of dogs, several studies have failed to find a difference in contagious yawning due to familiarity with individual people [14,18] and in empathetic-like behaviors toward distressed strangers [46,47], with dogs responding similarly to strangers and owners. Most studies of more complex prosocial behavior (e.g., trapped-other paradigms) in dogs have focused on the response of dogs to their owner’s distress [34,35,36,37,48]. Because familiarity bias might predict dogs would fail to help a stranger, it would be interesting to see whether dogs in a rescue paradigm would seek to help unfamiliar humans expressing distress. 

The present study sought to examine both the matching of others’ emotional states (emotional contagion) and prosociality components of empathy by measuring dogs’ door-opening behavior to a trapped stranger who was either in the distress condition (crying) or the neutral condition (humming). Measures of the dogs’ behavior and physiology (HRV) were also recorded during the session to assess emotional contagion between the human and the dog. Assessing both rescue behavior and emotional contagion measurements (i.e., HRV and stress behaviors) helps to assess the extent to which dogs respond to strangers in distress. By testing strangers rather than owners, the present study also allows empathy and prosocial behaviors to be tested with minimal desire for social contact with the unknown human, which has been a concern in past studies [49]. If dogs exhibit empathetic-like behaviors towards humans, we would expect a stronger response to the stranger in distress in physiology, behavior, and opening at a higher level compared to dogs in the neutral condition. This will help to further extend our knowledge about the evolution and existence of empathy in domestic dogs.

## 2. Materials and Methods

### 2.1. Animals

There were 35 household dogs, both male (N = 18) and female (N = 17) that were recruited and tested for this study. Only adult dogs participated in the study (*M* = 5.29 years, *SD* = 3.21, *Range* = 1–11). Owners volunteered their dogs for this study and were recruited by emails or from fliers. In the recruitment emails, dogs were assessed by their owners for their suitability to participate in the study by asking about comfort in novel locations and aggression towards people. Only dogs that were comfortable and non-aggressive were recruited. The sample of dogs contained mostly mixed-breed dogs (N = 20) and a variety of other purebred dogs (N = 15). The breeds represented by the 15 dogs were Golden Retrievers (3), Beagles (2), Labrador Retrievers (2), American Staffordshire Terrier (1), Chihuahuas (1), Corgi (1), Dachshund (1), Newfoundland (1), Standard Poodle (1), and Springer Spaniel (1). All methods were approved by the Ripon College IACUC (2018.05.Manor) and IRB (Manor2018).

### 2.2. Materials and Measures

The C-BARQ questionnaire was used to collect the owner-reported history of the dogs’ behaviors including aggression, anxiety, and attachment levels [50].

**Figure 1:**
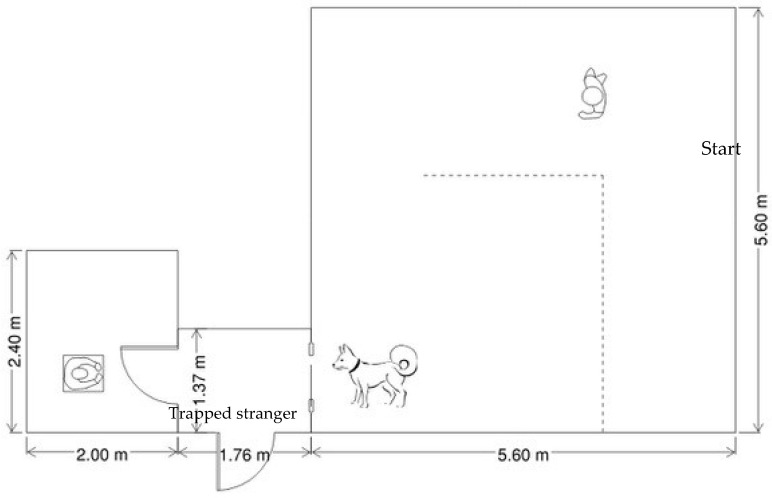
Schematic of room setup for experiment. Dotted line represents cloth barrier.

The heart rate variability of dogs was recorded using a Polar H7 Heart Rate Monitor (HRV; Polar Electro, Kempele, Finland). The data recorded from the heart monitor were transferred to an iPhone app via Bluetooth (Heart Rate Variability Logger App, A.S.M.A. B.V., https://apps.apple.com/us/app/heart-rate-variability-logger/id683984776 accessed on 20 June 2024). ARTIIFACT software (v.2.13) was used to analyze the heart rate variability data (Kaufman, T., https://github.com/tobias-kaufmann/ARTiiFACT accessed on 20 June 2024 [51]). A rectangular room with a small room adjacent to it was used as the testing arena (see Figure 1 for dimensions and setup). The larger room was broken into two parts with a cloth barrier dividing the room into two sections. In the small room, there was a chair placed in the middle of the room facing the testing door. The testing door was 96.5 cm wide × 122 cm tall × 2.54 cm thick with gray painted wood and clear plexiglass (55.9 cm × 106.7 cm) throughout the middle of the testing door to create a window. Three heavy-duty vertically placed magnets along the hinged side of the door were used to attach the testing door to the doorframe. Additionally, weaker vertically placed magnets were placed on the other side of the testing door to loosely connect the testing door to the metal doorframe. This allowed dogs to easily open the door by using their paw or nose as indicated by previous research [35]. 

Three cameras were set up to record the behaviors of dogs during the baseline and stranger prosocial tasks.

### 2.3. Procedure

One researcher, a female, was assigned to be the stranger throughout testing. The stranger was sitting in the smaller adjacent room with the door to the room closed and the testing door covered in black cloth while the dogs entered the testing room. Other researchers led the dog and the owner to the larger rectangular room, behind the room barrier, for the instructions and baseline measurements. There the researchers applied veterinary lubricant to the heart rate monitor, and the monitor was placed on the dog’s chest just to the left of center behind the dog’s front legs. Once a connection to the monitor was established, a baseline was recorded for 10 min while dogs were loosely held on their leash. This baseline included HRV measurements as well as coding for baseline stress behaviors, which were captured by the camera held by a researcher. During the baseline, owners completed the CBARQ survey online on a laptop computer next to their dog. Owners and experimenters were asked to ignore the dogs during this baseline measurement. Once the 10 min baseline had been completed, the owner left the room, accompanied by one researcher, out of sight of the dog. The exiting researcher uncovered and closed the testing door and opened the small adjacent room that contained the stranger. The researcher assigned to stay held onto the dog and waited for the start signal from the researcher who left. After the start signal was given, the researcher holding the dog in the baseline location (see Figure 1 for room schematic) unclipped the dog from their leash and the stranger-helping task began. 

The stranger-helping task used the same experimental task previously used with owners [35]. A between-subjects design was used to avoid the introduction of experience as an additional factor (as seen in previous research [37]) and to make the study as comparable as possible to Sanford et al. [35]. The dogs were randomly assigned to one of two conditions: humming or crying. In the crying conditions, the stranger in the adjacent room cried for 5 min while calling for help in a distressed voice every 15 s. In the humming condition, the stranger hummed “Twinkle Twinkle Little Star” and in a monotone voice said “help” every 15 s. The trial ended once the dog opened the door or after the 5 min had elapsed. Opening was defined as the dog initiating contact with the door causing the magnets to detach and the door to swing forward (see Supplemental Video S1 for opening example). Each trial was video recorded and then coded for stress behaviors and latency to open from the time of the start cue to the door opening. A latency of 300 s was recorded for dogs that did not open. The HRV of dogs was also recorded during the stranger prosocial task in both conditions.

### 2.4. Behavioral Ethogram

The behaviors of dogs were recorded with one camera during the 10 min baseline and from three cameras during the 5 min testing period. Panting and sniffing the floor were measured for the duration of time in seconds. The rest of the stress behaviors were recorded as the number of times the behavior was exhibited such as whining, barking, growling, urogenital checkouts, shake-offs, yawning, scratching, and tongue flicks ([52,53]; as used in [35]; see Table 1 for ethogram). All stress behaviors were summed and then divided by the number of seconds that the video was recorded for each dog to obtain the number of stress behaviors shown per second. This allowed for comparisons across videos of different lengths. Interrater reliability was measured for approximately 20% of the videos using a coder blind to the hypotheses. The correlation between coders was very high for both the baseline and the stranger prosocial task (*R*(5) = 0.99, *p* < 0.001, for each task).

Additionally, dogs were coded for the tone of their first approach to the door through which the stranger could be seen. These approaches were coded similarly to Custance and Mayer [46,47]. However, we added a sub-category to the coding to better capture the state of the dog (See Table 2 for a full description of each category). We subdivided the alert category into alert and alert + aggression. In the alert + aggression, dogs showed alert body posture but also barked or growled at the door during approach. Interrater reliability was also assessed for all dogs for tone of approach and agreement was 85% between raters.

### 2.5. Data Analysis

The C-BARQ survey compiles scores for different measures of attachment, fear, and aggression (e.g., fear of strangers, fear of dogs, non-social fear, dog-to-dog aggression, household dog aggression; [50]). These subscales were then summed to create a total attachment score, a total fear score, and a total aggression score. Of the 35 dogs tested, 1 dog was not included in the C-BARQ survey due to issues with the survey during data collection.

Regarding HRV measures, there were 4 dogs that were not measured for HRV because of an inability to gather a reading due to poor connectivity, primarily due to coat thickness. For baseline HRV, data were recorded for 10 min with the first minute being removed from analysis to ensure that good connectivity had been achieved. Inter-beat intervals over 2000 ms were labeled as artifacts in order to remove them from the data through cubic spline interpolation via the Artiifact software as performed in prior research [35]. The VLF, LF, and HF bands were set to 0.06 Hz, 0.24 Hz, and 1.06 Hz, respectively, as is congruent with previous canine heart rate measures [54]. Similar to previous studies, pNN50 was used as the primary measure of HRV due to its ability to work with differing time frames and the low impact of arrhythmias on the results [35,47,55].

Latency was not normally distributed based on Shapiro–Wilk tests so non-parametric tests were used. Two Mann–Whitney U-tests were used to test latency across conditions as well as latency across conditions only in dogs that opened to remove any ceiling effects from dogs that did not open (as in [35]). Because time near the door was normally distributed, we used an independent samples t-test to compare the proportion of time at the door by condition. A chi-squared was also run in order to test for any significant differences between the number of dogs that opened or did not open across conditions. Chi-squared tests were also used to measure the difference in tone of the door approach by condition and opening status. 

Although stress behaviors and owner-reported aggression were not normally distributed according to Shapiro–Wilk tests, we used ANOVAs to analyze the data as modeling has found that ANOVAs are extremely robust to violations of normality [56]. Two 2 × 2 × 2 mixed factorial ANOVAs were run to test for differences in time point (baseline vs. prosocial task), condition (crying vs. humming), and opening (openers vs. non-openers) on stress behaviors and separately on HRV. Additionally, a between-subjects 2 × 2 ANOVA compared owner-reported attachment, total fear, and aggression based on condition and opening status. 

Correlations were run in order to analyze relationships between owner-reported attachment, owner-reported fear, owner-reported aggression, stress behaviors in baseline and the stranger task, pNN50 measures, and the latency to open the door. Spearman’s correlations were run for comparisons involving latency, stress behaviors, and owner-reported aggression due to their lack of normality. Pearson’s correlations were run for comparisons that only involved pNN50 measures, owner-reported attachment, and owner-reported fear. 

## 3. Results

### 3.1. Stranger Prosocial Task

There was a total of 35 dogs that completed the experiment and a total of 15 dogs that opened across conditions. In the humming condition, eight (44%, N = 17) dogs opened the door for the stranger, while in the crying condition, seven (39%, N = 18) dogs opened. There was no significant difference between opening by condition, χ^2^(1, N = 35) = 0.24, *p* = 0.63. There were also no differences in the latency of dogs in the crying condition (*Med* = 300, *IQR* = 255.75) and humming condition (*Med* = 300, *IQR* = 273.5), *U* = 133.5, *z* = −0.71, *p* = 0.48. When only considering dogs that opened, there were no significant differences in latency to open in the humming condition (*Med* = 26.5, *IQR* = 73.75) and the crying condition (*Med* = 39, *IQR* = 27), *U* = 21, *z* = −0.81, *p* = 0.42. There were no sex differences in opening by the dogs, χ^2^(1, N = 35) = 0.38, *p* = 0.85, with females (~41%) and males (~44%) opening at similar rates. 

We also analyzed whether there were any differences in the proportion of time in the trial spent in proximity to the door by condition. Dogs in the humming condition spent approximately the same proportion of time by the door (*M =* 0.34, *SD* = 0.25) as the crying condition (*M =* 0.37, *SD* = 0.27), *t*(32) = −0.623, *p* = 0.54. 

### 3.2. Physiological and Behavioral Measures

Dogs’ HRV did not appear to differ significantly by status or by condition (All *F*’s < 1.8, *p* > 0.10). Time point, however, was significant. Dogs, regardless of condition and opening status, exhibited significantly more stress behaviors during the baseline (*M* = 0.38, *SD* = 0.26) than during the distress and neutral condition trials (*M* = 0.19, *SD* = 0.18), *F* (1, 30) = 13.17, *p* = 0.001, *η*^2^ = 0.31. There were no other main effects or interactions that were significant (All *F’s <* 1.0, *p’s* > 0.10).

**Figure 2:**
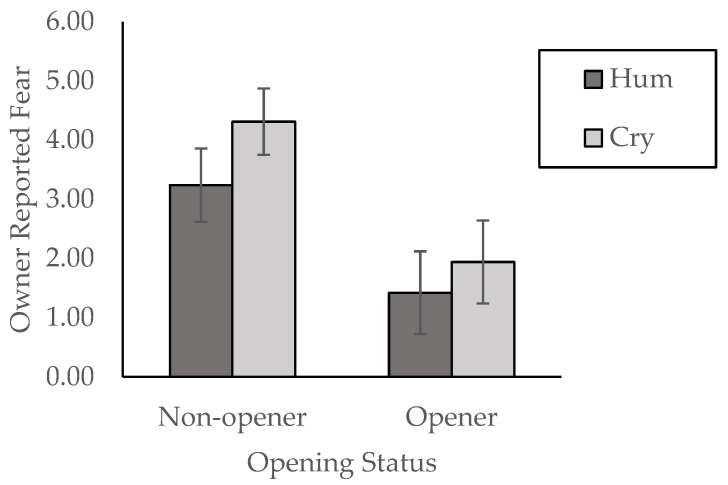
The average owner-reported fear in the dog compared by condition and opening status. Error bars represent the standard error of the mean.

For owner-reported fear, there was a significant main effect of opening found, with openers having significantly lower owner-reported fear (*M* = 1.68, *SD* = 1.18) than dogs that did not open (*M* = 3.83, *SD* = 2.12), *F* (1, 30) = 10.43, *p* = 0.003, *η*^2^ = 0.26 (see Figure 2). There were no other significant main effects or interactions (All *F’s <* 1.6, *p’s* > 0.10). There were no significant differences in owner-reported aggression across any groups (All *F*’s < 2.0, *p’s* > 0.10) or in owner-reported attachment across any groups (All *F*’s < 2.31, *p’s* > 0.10).

**Figure 3:**
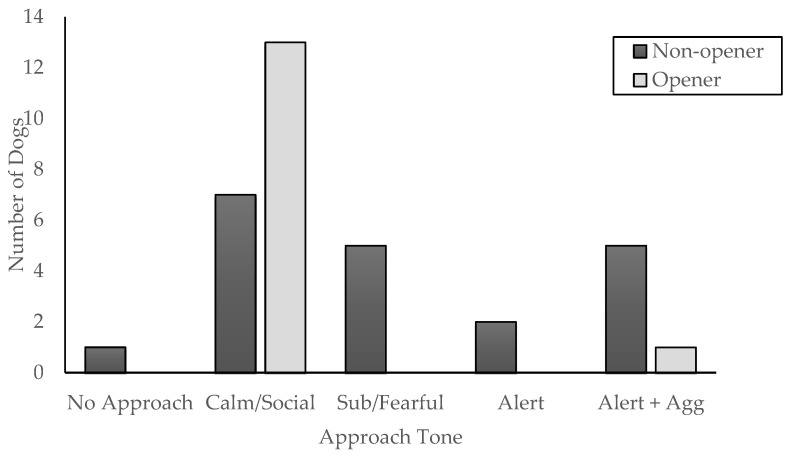
Tone of approach for the first approach to the door that held the stranger presented by opening status.

One of the dogs that opened the door could not be coded for the first approach as the video did not show the approach clearly for that dog. The chi-squared tests on the tone of approach found that the tone of approach only differed by opening status, χ^2^(5, N = 35) = 11.77, *p* = 0.019. Dogs that opened the door were primarily calm/social (N = 13; 92%), with only a single dog showing other behavior (alert + aggressive; 8%). By contrast, dogs that did not open showed more varied behavior but were also primarily calm/social (N = 7, 35%), followed by alert + aggressive (N = 5, 25%) and submissive-fearful (N = 5, 25%). There were also two dogs who were alert (10%) and one dog that never approached the door (5%) (See Figure 3). There was no difference in the tone of approach by crying or humming condition, χ^2^(5, N = 35) = 3.09, *p* = 0.54.

### 3.3. Correlations 

Total owner-reported aggression and total owner-reported attachment were not significantly predictive with any empathy measures so they were not included in the table (All *Rs* < 0.30, *p’s* > 0.10).

As seen in Table 3, a significant positive correlation was found between latency to open and owner-reported fear in the crying condition but not in the humming condition. There was also a positive correlation between the pNN50 HRV during baseline and pNN50 HRV during the stranger trials of dogs in the crying condition, and a marginally significant correlation was found in dogs in the humming condition. In both conditions, there was a significant negative correlation between the number of stress behaviors exhibited during the baseline and the baseline pNN50 HRV.

Interestingly, when only openers were considered in the latency measure, there was a strong negative correlation between latency to open and the dog’s pNN50 HRV during that stranger opening for both the humming (*R* = −0.77, *p* = 0.045) and crying (*R* = −0.82, *p* = 0.048) groups. Dogs with higher HRV and lower stress showed quicker opening latencies. 

## 4. Discussion

Dogs were willing to open the door for the trapped individual in both conditions (approximately 42% of dogs opened), as found in previous research [35,36,37]. However, there was no difference in the number of dogs opening between the distress and neutral conditions as in Sanford et al. [35] but in contrast with Carballo et al. [36] and Bourg et al. [37]. Both Carballo et al. [36] and Bourg et al. [37] used restraining apparatuses in which the human was trapped in a box that the dog had to open by swinging the door outward. In Sanford et al. [35] and the present study, dogs opened a door to an adjacent room by pushing on an inward swinging door. The differences in ease of opening and distance from the human may account for the higher rates of opening. Perhaps the opening task was too easy to show differentiation by emotional state (see the argument in [37]); however, we feel that an easier opening mechanism better illustrates dogs’ intentions without as many limitations based on abilities that other studies have found [37]. It is possible, though, that a more difficult mechanism requires the dog to stop and think more which may produce differences by condition. 

This experiment suggests that dogs open the door for reasons other than social contact. During the stranger prosocial task, an experimenter was located in the larger room recording the trial. If the opening of the door was due to a desire for social contact, it is not clear why the dogs worked to open a door to access the novel human rather than maintaining contact with the human who was already accessible. However, it is likely that the noise of the stranger, crying or humming, could have produced stimulus enhancement effects for door opening in this experiment. 

Unlike Sanford et al. [35], we found no significant difference in latency to open based on the condition. This was driven both by the quicker latency in the crying condition for Sanford (*M =* 23.43) and the slower latency in their humming condition (*M =* 95.89) compared to our slower latency to open in crying (*M =* 38.29) and faster latency in humming (*M =* 48.88). These differences may be due to differences in the experimental set-up or room design between the two studies. However, it may also make sense for dogs to be more cautious when opening a door for a stranger, which may explain the slower latency in the crying condition. It is not clear why the humming condition would not also have shown that caution and instead have been faster than with owners. This may have been due primarily to chance as the small number of dogs in each condition opening could have skewed the opening times in the present study and/or in Sanford’s. It is interesting to note that in both studies, the variability within the latency to open was much greater in the humming condition than in the crying condition. This may suggest that dogs have more varied motivations or less urgency for opening in the humming condition compared to the crying condition. It is possible that dogs were unable to discriminate between the emotional states of the stranger, which led to the lack of difference in responding in the present study. It is unclear though why dogs would be unable to discriminate the stranger’s emotional states when, in previous studies, dogs have had no difficulty [46,47].

Dogs showed a somewhat different pattern of stress responses in the present study than in Sanford et al. [35]. As in Sanford, dogs showed a positive correlation between owner-reported fear and latency to open in the crying condition (*R*(16) = 0.54 vs. Sanford *R*(15) = 0.48), suggesting that tendencies toward anxiety inhibited action in the stranger prosocial task. This result has also been found in studies of empathy in children [57,58]. Whereas in Sanford’s study, there was a non-significant negative correlation between owner-reported anxiety and latency to open in the humming condition (*R*(15) = −0.39), the current study found a marginally significant positive correlation (*R*(15) = 0.44). This pattern can also be seen in the ANOVA analysis of owner-reported fear. Thus, when owners were trapped in Sanford et al., dogs tended to open more in the control condition if they were anxious but less in the crying condition; while for strangers, anxious dogs were less likely to open in both conditions. High stress in animals seems to inhibit empathy responses in rats [59] as well as humans and mice [60]. Strangers, in the absence of the owner, are likely to induce stress responses in dogs [61,62], particularly dogs who are already more anxious. Additionally, strangers are unlikely to provide comfort to anxious dogs, which may explain the different patterns of responses compared to owners [49]; however, there can be breed differences in tendencies to seek out owners over strangers [63]. We did not have sufficient breed representations to analyze differences in patterns of opening by breed, but this may be an area to consider in future research. We did not collect information about training backgrounds in the present study. Although Sanford et al. [35] did not find any differences between dogs that were specifically trained for attention to people (therapy dogs), it is possible that general training relates to familiarity and comfort with strangers. It would be beneficial for future research to record training class backgrounds to allow for comparisons to be made with behavior toward a stranger.

Interestingly, our dogs did not show an increase in stress when confronted with the trapped stranger but instead showed decreased stress behaviors and a stable pNN50 (HRV). This differs from past research that has found the prosocial task more stressful based on heart rate [36], HRV [35], or behavior [37], particularly in the distress condition. The novelty of the environment and experimenters in the baseline appear to have been more stressful than the stranger trapped behind the door. This was the same pattern as openers showed in Sanford’s study, but the opposite pattern of non-openers. An inability to access the owner, whether distressed or not, appears to have distressed the dogs more than an inability to access a stranger in the present study. It is possible that the higher level of stress during the baseline in the present experiment was due to the dogs being on leash; however, the baseline stress behaviors were similar to Sanford et al. [35] (approximately 0.4 behaviors/second in the baseline), but future research may wish to run baseline measures off leash when possible.

Many dogs in the present study, particularly the non-openers, approached the stranger with fear and aggression as evident in the significant differences in tone of approach to the stranger between openers and non-openers. The aggression was primarily seen in dogs who did not open the door. These approaches to the door often involved barking, which was never seen in the owner prosocial task. In our study, openers tended to approach the door with calm and social postures. Strangers may induce stress, which makes emotional contagion less likely to occur [60], which may explain the lack of emotional differences in the dogs by condition. Studies in dogs’ households with the owner present have not found differences in empathetic-like responses to owners and strangers in distress [46,47]. However, an owner’s presence moderates stress responses to a stranger [62], which may explain the different results found in the current study. Familiarity with their owner may also allow dogs to identify visual and auditory cues of distress more accurately than with a stranger [64], which may make empathy more likely toward owners.

Overall, it is still unclear whether or not rescue behavior for an individual in distress can be considered as truly empathetic behavior (see argument in [65]). In the current study, there is no evidence of prosocial empathetic behaviors towards the stranger in distress or of any emotional contagion exhibited by dogs during the distress condition. The lack of evidence for empathy creates two possible scenarios as to why dogs may open the door. On the one hand, it is possible that dogs opening the door for their owners in distress more quickly or more frequently is a sign of empathy. Therefore, it is possible that the reason why no empathetic behaviors were recorded in this study is that unfamiliar humans in distress do not motivate dogs to exhibit empathy, as predicted by familiarity bias [15]. The other possibility is that dogs opened for their owners merely as a means of social interaction or to gain contact due to past learning about interactions with humans in distress. In fact, Bourg et al. [37] found that past experiences with door opening were a major contributor to differences in opening between dogs. In order to determine the mechanisms behind empathy in dogs, we think that more studies should be performed examining responses to trapped strangers before a conclusion is made. Dogs may view strangers with curiosity and thus approach them equally in the humming and distress conditions. This approach to rescue the stranger seems to be determined primarily by lower levels of anxiety in the dog. 

In order to continue to investigate the motivation behind rescuing an individual, future research should continue to examine the role of familiarity in empathy by using individuals known to the dog but not in a close relationship. It would also be interesting to test sociality towards strangers prior to the rescue task to see whether that would allow the prediction of which dogs would open. Research should also examine the role of task difficulty in our ability to see evidence of empathetic behavior. Additionally, tests correlating different measures of empathy and emotional contagion toward a stranger or their owner could be performed to better understand the mechanisms of prosocial and empathetic behavior. 

## 5. Conclusions

Empathy is a complex cognitive function that may have been impacted by the coevolution of humans and dogs [9]. Anecdotal evidence from across the world has shown that dogs can help a human in need; however, it is possible that these dogs are the exception rather than the rule. The current study could not provide evidence of empathy towards a stranger in distress; however, the limitations of the current study suggest further research is warranted.

## Figures and Tables

**Table 1:** Ethogram for coding the stress behaviors during baseline and prosocial tasks.

Category	Posture
Panting	Mouth open and tongue out rhythmic heavy breathing (in seconds)
Sniffing Floor	Head down to floor while breathing in through the nose (in seconds)
Whining	High-pitched repetitive vocalization
Barking	Low-pitched and short duration vocalization
Growling	Low-pitched and rumbling vocalization
Urogenital checkouts	Smelling urogenital areas
Shake offs	Motions of rotation in the body and/or head
Yawning	Opens mouth and inhales
Scratching	Using the paw to make repeated contact to the body or face
Tongue flicks	Tongue extends out of mouth and flicks nose

**Table 2:** Ethogram for coding the tone of approach for the dog’s initial door approach during the prosocial task.

Category	Posture
Calm/Social	Ears neutral, mouth open and relaxed, body relaxed, tail neutral to wagging
Submissive/Fearful	Ears back, mouth closed, body crouching and small, tail tucked
Alert	Ears up, mouth closed, body stiff, tail up
Alert + Aggressive	Same as alert, but with the addition of growling or barking
Playful	Ears up or neutral, mouth open, body in play bow (on elbows), tail up

**Table 3:** Summary of correlations, means, and standard deviations of dog’s distress measures, owner-reported fear, and latency to open.

	M	SD	1	2	3	4	5
Crying							
Latency to Open (s)	198.22	131.61					
2. Owner Reported Fear	3.39	2.23	0.55 *				
3. Baseline Stress Behaviors	0.40	0.29	0.03	−0.10			
4. Stranger Stress Behaviors	0.19	0.21	0.20	−0.001	0.37		
5. Baseline pNN50	47.04	21.61	−0.15	−17	−0.63 *	−0.23	
6. Stranger pNN50	46.09	21.97	−0.36	0.02	−0.11	−0.08	0.54 *
Humming							
Latency to open	181.64	134.26					
2. Owner Reported Fear	2.44	1.93	0.39				
3. Baseline Stress Behaviors	0.35	0.24	0.03	−0.26			
4. Stranger Stress Behaviors	0.18	0.14	0.09	0.17	−0.03		
5. Baseline pNN50	42.13	16.03	−0.22	−0.36	−0.65 *	−0.44	
6. Stranger pNN50	40.95	15.67	−0.16	−0.03	−0.31	−0.22	0.47 +

Note: + *p* < 0.10, * *p* < 0.05.

## Data Availability

The original data presented in the study are openly available in Open Science Framework at https://osf.io/5zkxe/.

## Supplementary Materials

The following supporting information can be downloaded at: https://www.mdpi.com/article/10.3390/ani14142130/s1, Video S1: Dog opening door.
